# Auxin response factors: important keys for understanding regulatory mechanisms of fleshy fruit development and ripening

**DOI:** 10.1093/hr/uhae209

**Published:** 2024-07-31

**Authors:** Bai-Jun Li, Ruo-Xuan Bao, Yan-Na Shi, Donald Grierson, Kun-Song Chen

**Affiliations:** State Key Laboratory for Conservation and Utilization of Subtropical Agro-Bioresources, College of Agriculture, Guangxi University, No.100, East Daxue Road, Xixiangtang District, Nanning, Guangxi 530004, China; State Agriculture Ministry Laboratory of Horticultural Plant Growth, Development and Quality Improvement, Zhejiang University, Zijingang Campus, No. 866, Yuhangtang Road, Xihu District, Hangzhou, 310058, China; Zhejiang Key Laboratory of Horticultural Crop Quality Improvement, Zhejiang University, Zijingang Campus, No. 866, Yuhangtang Road, Xihu District, Hangzhou 310058, China; State Key Laboratory for Conservation and Utilization of Subtropical Agro-Bioresources, College of Agriculture, Guangxi University, No.100, East Daxue Road, Xixiangtang District, Nanning, Guangxi 530004, China; State Agriculture Ministry Laboratory of Horticultural Plant Growth, Development and Quality Improvement, Zhejiang University, Zijingang Campus, No. 866, Yuhangtang Road, Xihu District, Hangzhou, 310058, China; Zhejiang Key Laboratory of Horticultural Crop Quality Improvement, Zhejiang University, Zijingang Campus, No. 866, Yuhangtang Road, Xihu District, Hangzhou 310058, China; College of Agriculture and Biotechnology, Zhejiang University, Zijingang Campus, No. 866, Yuhangtang Road, Xihu District, Hangzhou 310058, China; State Agriculture Ministry Laboratory of Horticultural Plant Growth, Development and Quality Improvement, Zhejiang University, Zijingang Campus, No. 866, Yuhangtang Road, Xihu District, Hangzhou, 310058, China; Division of Plant and Crop Sciences, School of Biosciences, University of Nottingham, Sutton Bonington Campus, Loughborough LE12 5RD, UK; State Agriculture Ministry Laboratory of Horticultural Plant Growth, Development and Quality Improvement, Zhejiang University, Zijingang Campus, No. 866, Yuhangtang Road, Xihu District, Hangzhou, 310058, China; Zhejiang Key Laboratory of Horticultural Crop Quality Improvement, Zhejiang University, Zijingang Campus, No. 866, Yuhangtang Road, Xihu District, Hangzhou 310058, China; College of Agriculture and Biotechnology, Zhejiang University, Zijingang Campus, No. 866, Yuhangtang Road, Xihu District, Hangzhou 310058, China

## Abstract

Auxin response transcription factors (ARFs) form a large gene family, many of whose members operate at the final step of the auxin signaling pathway. ARFs participate directly in many aspects of plant growth and development. Here we summarize recent advances in understanding the roles of ARFs in regulating aspects of fleshy fruit development and ripening. ARFs play a crucial role in regulating fruit size, color, nutrients, texture, yield, and other properties that ultimately influence the ripening and quality of important crops such as tomato, apple, strawberry, and peach. ARFs impact these processes acting as positive, negative, or bidirectional regulators via phytohormone-dependent or -independent mechanisms. In the phytohormone-dependent pathway, ARFs act as a central hub linking interactions with multiple phytohormones generating diverse effects. The three domains within ARFs, namely the DNA-binding domain, the middle region, and the carboxy-terminal dimerization domain, exhibit distinct yet overlapping functions, contributing to a range of mechanisms mediated by ARFs. These findings not only provide a profound understanding of ARF functions, but also raise new questions. Further exploration can lead to a more comprehensive understanding of the regulatory mechanisms of fleshy fruit development and ripening mediated by ARFs.

## Introduction

Fleshy fruits are popular worldwide and make important contributions to human health and the economy. Fruit development and ripening involve a series of complex physiological and biochemical processes that determine quality, yield, and nutritional value. Fruit development normally begins with fertilization, which stimulates the ovary and/or other floral parts to develop into a fleshy fruit, although fruit set and parthenocarpy can also occur naturally, or be induced by supplying phytohormones, without fertilization. During seed development, the fruit accumulates stored reserves and remains unattractive to eat. At maturity, the ripening process involves changes in color, flavor, texture, and aroma, which contribute to formation of quality attributes and impact attractiveness, nutritional value and storage life [1]. Hence, the regulatory mechanisms underlying the ripening processes are important research hotspots.

**Figure 1:**
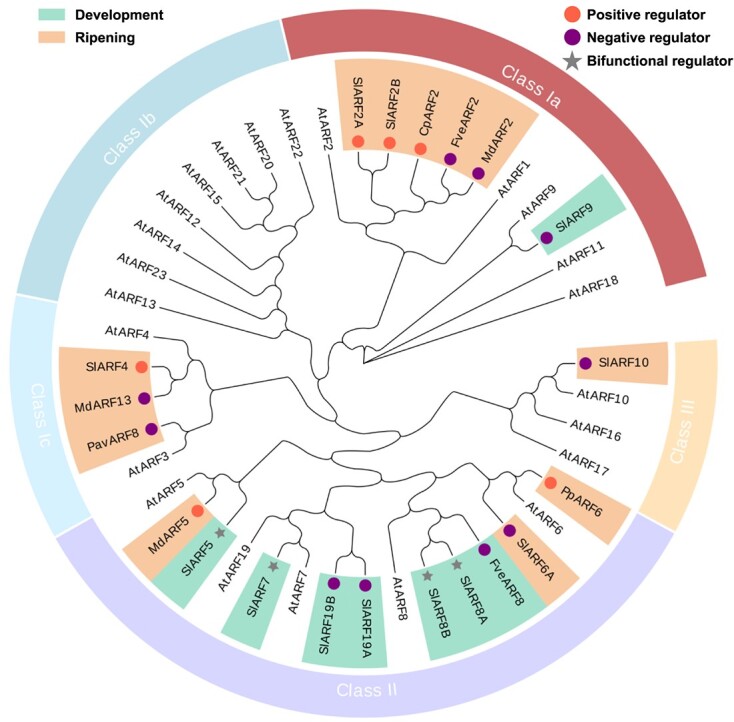
Phylogenetic tree of ARFs involved in fleshy fruit development or ripening. Amino acid sequences of ARFs verified to participate in fleshy fruit development or ripening and ARF family members from *Arabidopsis thaliana* were used to construct a phylogenetic tree based on neighbor-joining. The sequence alignments were performed with MUSCLE. Green and orange rectangles represent genes involved in regulating fruit development and ripening, respectively. Red and purple circles in rectangles indicate genes with positive and negative roles, respectively, controlling fruit development or ripening, and gray stars denote bidirectional regulators performing both positive and negative functions in the processes. The accession numbers of ARF proteins used in this analysis are as follows: for AtARFs from *A. thaliana* refer to the previous study [2]; SlARF2A (NP_001233765.1), SlARF2B (NP_001233765.1), SlARF4 (NM_001246842.2), SlARF10 (NM_001247867.2), PavARF8 (XP_021830228.1), and FveARF2 (XP_004297494.1) from NCBI GeneBank; MdARF2 (HF41569), MdARF5 (MDP0000211459), and MdARF13 (HF40493) from Genome Database for Rosaceae (https://www.rosaceae.org/); FveARF8 (FvH4_1g27650) and PpARF6 (Prupe.4G085900) from Phytozome (https://phytozome-next.jgi.doe.gov/); SlARF5 (Solyc04g084210), SlARF7 (Solyc07g042260), SlARF8A (Solyc03g031970), SlARF8B (Solyc02g037530), SlARF9 (Solyc08g082630), SlARF19A (Solyc07g01618), and SlARF19B (Solyc05g047460) from Sol Genomics Network (https://solgenomics.net/search); CpARF2 [3] and SlARF6A [4] from their supplementary Table S1 and Figure S1, respectively. The ARFs shown are from apple (Md), tomato (Sl), woodland strawberry (Fve), peach (Pp), sweet cherry (Pav), papaya (Cp), and arabidopsis (At).

Auxin is an important phytohormone that participates in controlling fleshy fruit development and ripening via its signaling pathway [2]. Auxin signaling involves its direct binding to TIR1/AFB (TRANSPORT INHIBITOR RESISTANT 1/AUXIN SIGNALING F-BOX), which can recruit other components to form an SCF ubiquitin protein ligase, which causes the degradation of the inhibitory Aux/IAA (AUXIN/INDOLE-3-ACETIC ACID) protein by the ubiquitin-proteasome system [3, 4]. Subsequently, the ARFs (auxin response factors), previously constrained by Aux/IAA, are released and can function in the regulation of auxin-dependent gene expression [5]. Therefore, ARFs are regarded as an indispensable connecting link between the auxin signaling pathway and the various downstream response processes triggered by auxin [6].

It is well known that ARFs play a crucial role in various aspects of plant growth and development [7, 8], including fruit development and ripening. These key transcription factors usually possess three important domains, comprising a conserved B3-type DNA binding domain (DBD) at their N-terminus, a middle region (MR) following the DBD, and a carboxy-terminal dimerization domain (CTD: domain III/IV, also named PB1 or AUX/IAA) at the C-terminus [9]. The DBD domain can bind to the auxin response element (AuxRE, TGTCTC/GAGACA) in the promoter region of target genes to regulate their expression [10, 11], and the MR region determines whether ARFs act as inhibitors or activators of the expressions of downstream genes [12, 13], while the CTD is involved in the interaction with Aux/IAA to control the ARF activity [14]. These three domains determine the functions of ARFs in the phytohormone-dependent processes. In recent years, more and more studies have shown that auxin and other phytohormones influence fleshy fruit development and ripening via ARFs [2, 15], and they are an attractive exploratory node that connects auxin to the regulation of these two processes.

This review summarizes the current developments in understanding of the roles of ARFs in fleshy fruit development and ripening and the phytohormones they respond to, and also discusses future perspectives in this important research field.

## Three roles of auxin response factors in fleshy fruit development

**Figure 2:**
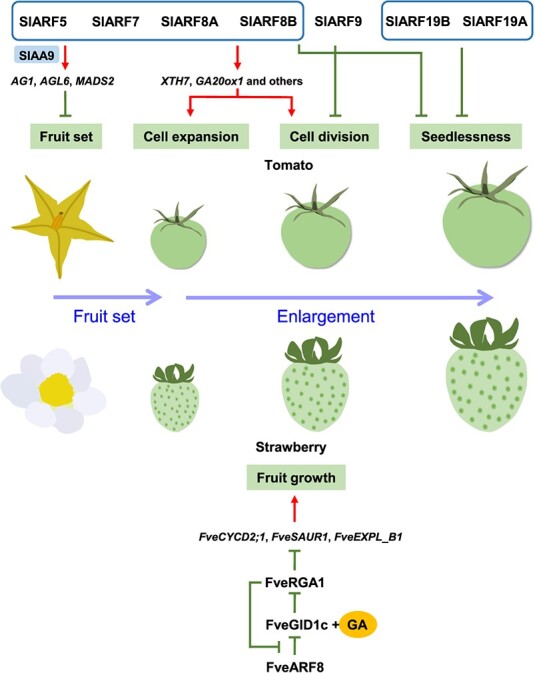
Roles of ARFs in fleshy fruit development. The deduced roles of ARFs reported to participate in fleshy fruit development in tomato and strawberry are shown. Red arrows and green lines indicate activation and inhibition, respectively. ARF, auxin response factor; IAA, AUX/IAA protein; AG, AGAMOUS1; AGL, AGAMOUS-LIKE 6; MADS, MADS-BOX; XTH, xyloglucan endotransglucosylase/hydrolase; GA20ox, GA 20-OXIDASE; GID, GIBBERELLIN INSENSITIVE DWARF; GA, gibberellin; RGA, DELLA protein; CYCD, Cyclin D; SAUR, small auxin upregulated RNA; EXP, EXPANSIN. Based on research discussed in the text.

During fleshy fruit development after pollination, the fruit enlarges to the maximal size accompanied by tissue differentiation, cell division and/or expansion, and deposition of reserves, during which auxin usually plays an important role [2]. The functions of ARFs in this process have been widely reported for tomato (*Solanum lycopersicum*), a model climacteric fruit. Cell expansion and division are very important phases to determine fruit size [16, 17]. Early investigations showed there are three *SlARF*s involved in these processes. SlARF9 (Fig. 1) has been found to negatively control cell division and tomato fruit size based on the results of overexpression and RNA interference (RNAi) experiments to probe their functions [18], but further exploration of its specific mechanism is required (Fig. 2). The tomato fruit of *SlARF5*-supressed lines generated by RNAi were smaller than the wild type (WT), and their pericarps showed a significant decrease in cell layers and a larger cell size compared to the WT [19]. Therefore, SlARF5 plays a positive and negative role in cell division and cell expansion, respectively. Moreover, the fruits of *SlARF7*-suppressed lines produced by RNAi had a remarkable heart-like shape and there was no significant difference in the size compared with the WT, but their pericarps were thicker than WT [20]. Microscopic examinations indicated that this difference between the pericarps was caused by an increase in cell expansion and there was no visible difference in cell division, suggesting SlARF7 acted as an inhibitor of cell expansion. Furthermore, both *SlARF5*- and *SlARF7*-suppressed lines could generate parthenocarpic fruits after the flowers had been emasculated, further confirming these two *SlARF*s participated in fruit growth [19, 20].

However, Hu *et al*. [21] found that multiple *SlARF*s, including *SlARF5*, *SlARF7*, and *SlARF8B*, were downregulated in *SlARF7*-RNAi lines, suggesting that inhibiting expression of multiple *SlARF*s resulted in the parthenocarpic phenotype of the *SlARF7*-RNAi lines. The *slarf7* or *slarf5* single mutant generated by CRISPR-Cas9 did not produce parthenocarpic fruits after emasculation, while the *slarf7 slarf5* double mutant could undergo parthenocarpy, indicating the heart-like shape and parthenocarpic fruits of the *SlARF7*-RNAi lines [20] might be caused by decreasing the expressions of multiple *SlARF*s. Therefore, this situation may also exist in the *SlARF5*-RNAi lines [19]. Additionally, SlARF7 has a regulatory bidirectional effect (i.e. both positive and negative effects) on tomato fruit development by controlling fruit growth-related genes (e.g. *EXPANSIN5*) and is a key point in the crosstalk between auxin and gibberellin (GA) [21]. In this process, AUX/IAA protein 9 (SlIAA9, which inhibits auxin signaling) and SlDELLA (a GA signaling factor) interact with SlARF7 to regulate the ethylene biosynthetic gene (*ACC oxidase4*, *ACO4*) and the feedback-regulated genes involved in GA (*GA 20-OXIDASE 1* and *GA 3-OXIDASE 1*, i.e. *GA20ox1* and *GA3ox1*) and auxin (*auxin responsive GH3.2*, i.e. *GH3.2*) metabolism before pollination, which may result in promoting ethylene production and inhibition of GA and auxin accumulation to suppress fruit initiation and growth. Upon pollination, there are increases in auxin and GA levels that induce SlIAA9 and SlDELLA degradation to release SlARF7, thus positively regulating fruit set and growth. Besides SlARF7, five other SlARFs (i.e. SlARF5, SlARF8A, SlARF8B, SlARF19A, and SlARF19B) also can interact with SlIAA9 and SLDELLA, and it is hypothesized that SlARF7 and additional SlARFs play overlapping roles in managing tomato fruit development [21].

Recently, the fruits of tomatoes with *SlARF8A* or *SlARF8B* knocked out by CRISPR-Cas9 were shown to be smaller than WT, and the fruits of the double mutant were smaller than those of any single mutant [22]. Moreover, the single mutants of *slarf8a* or *slarf8b* and the *slarf8a slarf8b* double mutant could produce parthenocarpic tomato fruits after emasculation, and the fruit size of the *slarf8a slarf8b* double mutant was significantly bigger compared with any single mutant. These results indicate that SlARF8A and SlARF8B act as negative regulators for fruit initiation and positive manipulators for fruit growth. In addition to SlARF8A and SlARF8B, another two class A ARFs, SlARF5 and SlARF7, have also been verified to be bidirectional regulators in tomato fruit development [22] (Fig. 1). These four SlARFs interact with SlIAA9 to repress fruit initiation via activating the expression of *MADS-BOX* (*MADS*) genes, including *AGAMOUS1* (*SlAG1*), *AGAMOUS-LIKE 6* (*SlAGL6*), and *SlMADS2*, and also enhance the expressions of *Xyloglucan endotransglucosylase/hydrolase 7* (*XTH7*), *GA20ox1* and others without SlIAA9 to promote fruit growth [22], which is supported by the results from another study in the same year [23] (Fig. 2). Additionally, the single mutants of *slarf8a* or *slarf8b* produced by CRISPR-Cas9 had an increased percentage of seedless fruits compared with WT, while all of the self-fertilized fruits generated from the *slarf8a slarf8b* double mutant were seedless [23]. Moreover, several *slarf8* mutant combinations could increase fruit yield under extreme temperatures (i.e. under 34°C day/28°C night temperatures) [23], indicating that these mutants and *SlARF8*s may be utilized in breeding to enhance yields of tomato and other fleshy fruits via crossbreeding and transgenic technologies. Notably, when *SlARF19A* and *SlARF19B*, which have a close sequence similarity to *SlARF7* (Fig. 1), were used to produce a double mutant generated by CRISPR-Cas9, the tomato fruits produced by self-pollination were seedless [23]. They can also interact with SlIAA9 and SLDELLA proteins [21]. These findings suggest that *SlARF19A* and *SlARF19B* may have a similar function to the four class A *SlARF*s in tomato fruit development (Fig. 2), however, the mechanism remains unclear.

There are fewer reports on the functions of ARFs in other fleshy fruits, probably due to lack of rapid functional transgenic systems. Recently, the function of *FveARF8* (Fig. 1) has been characterized in diploid strawberry (*Fragaria vesca*), a model non-climacteric fruit. The *arf8* mutant lines generated by CRISPR-Cas9 develop larger and rounder fruits compared with WT but are unable to produce parthenocarpic fruits after emasculation, indicating that *FveARF8* serves as a negative regulator for fruit growth and does not impact fruit initiation [26]. This suggests that the function of *SlARF8*s in climacteric fruit growth [22, 23] is opposite to that of *FveARF8* in non-climacteric fruit [26]. The mechanism, mediated by FveARF8, has been found to involve a link in the interaction between auxin and GA in strawberry fruit growth, where a DELLA protein, FveRGA1, interacts with FveARF8 to repress the inhibitory effect of FveARF8 on the expression of the GA receptor *GIBBERELLIN INSENSITIVE DWARF1c* (*FveGID1c*; Fig. 2). FveRGA1 has been found to repress fruit growth by downregulating *Cyclin D 2;1* (*FveCYCD2;1*), *small auxin up-regulated RNA 1* (*FveSAUR1*), and *EXPANSIN L_B1* (*FveEXPL_B1*) expression, suggesting it plays an important role in cell expansion and division during fruit development [26]. Moreover, FveGID1c plus GA can directly interact with FveRGA1 to degrade the latter. This suggests a feedback regulation model where FveRGA1-FveARF8-FveGID1c maintains FveRGA1 at a high and low level at pre- and post-fertilization, respectively, resulting in promotion of fruit development after post-fertilization [26].

## ARFs are positive or negative regulators of fleshy fruit ripening

**Figure 3:**
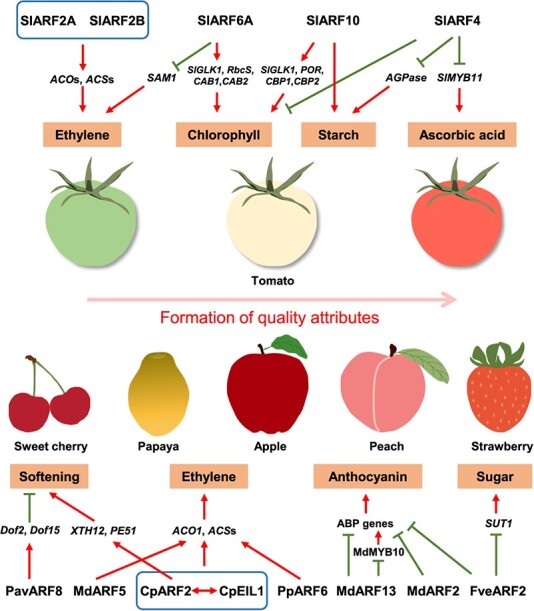
Roles of ARFs in formation of quality attributes during fleshy fruit development and ripening. Red arrows and green lines indicate promotion and inhibition, respectively. ARF, auxin response factor; ACO, ACC oxidases; ACS, ACC synthases; SAM, *S*-adenosylmethionine synthetase; GLK, GOLDEN2-LIKE; RbcS, ribulose bisphosphate carboxylase small chain; CAB, chlorophyll A/B binding protein; POR, protochlorophyllide oxidoreductase; CBP, chlorophyll binding protein; AGPase, ADP-Glc pyrophosphorylase; Dof, DNA-binding with one finger; XTH, xyloglucan endotransglucosylase/hydrolase; PE, pectin methylesterase; ABP, anthocyanin biosynthesis pathway; SUT, sucrose transporter gene. Genes are from apple (Md), tomato (Sl), woodland strawberry (Fve), peach (Pp), sweet cherry (Pav), and papaya (Cp).

Fleshy fruit qualities, such as aroma, texture, flavor, and color, contribute to their commercial value and consumer satisfaction and are formed during ripening [27]. ARFs have been found to not only participate in fruit development but also be activators or inhibitors of ripening. In tomato fruit, the expression levels of *SlARF2A* and *SlARF2B* increase during ripening, and *SlARF2A* expression responds to ethylene while *SlARF2B* responds to auxin [28]. Silencing *SlARF2A* or *SlARF2B* by RNAi inhibits tomato fruit ripening and the *SlARF2*s double suppressed-lines show lower levels of expression of *ACC oxidase*s (*ACO*s) and *ACC synthase*s (*ACS*s), encoding ethylene biosynthetic enzymes. The reduced ethylene production and more severe repression of ripening shown by these fruits indicate that these two *SlARF2*s act as ripening activators and there is a functional redundancy between them (Fig. 3). Additionally, *SlARF2A*-overexpressing lines show reduced accumulation of salicylic acid (SA) and abscisic acid (ABA) and enhanced ethylene and cytokinin (CTK) biosynthesis, suggesting that SlARF2A influences interactions between phytohormones in tomato ripening [29]. Jones *et al*. [30] found that transcripts of *DEVELOPMENTALLY REGULATED12* (*DR12*), an *ARF* gene now named *SlARF4* (Fig. 1), increased with tomato fruit ripening. Downregulating *SlARF4* in tomatoes using sense or antisense gene suppression resulted in a significant increase in chloroplast number that generated a dark-green fruit phenotype, and *SlARF4*-inhibited lines showed blotchy ripening fruit [30]. Further, *SlARF4* has been found to negatively regulate starch biosynthesis, especially genes coding for ADP-Glc pyrophosphorylase (AGPase), sugar metabolism, and chlorophyll accumulation [31]. These results strongly suggest that *SlARF4* plays a positive role in tomato ripening (Fig. 3). Recently, SlARF4 has been found to be a negative regulator of ascorbic acid (AsA, i.e. vitamin C) biosynthesis in the tomato fruit, which is mediated by an auxin and abscisic acid (ABA) antagonistic mechanism, where the gene encoding mitogen-activated protein kinase 8 (SlMAPK8) is upregulated by ABA, resulting in phosphorylation of SlARF4 to repress its transcriptional repression of *SlMYB11*, a promoter of AsA accumulation [32] (Fig. 3). On the other hand, SlARF6A [25] and SlARF10 [33] have been verified as activators of sugar and chlorophyll accumulation and repressors of fruit ripening. The mechanism involves SlARF6A directly binding to the promoters of *GOLDEN2-LIKE 1* (*SlGLK1*), *chlorophyll A/B binding protein*s (*CAB*s), and *ribulose bisphosphate carboxylase small chain* (*RbcS*), to promote sugar via positively regulating *SlGLK1* and chlorophyll accumulation via positively controlling *CAB*s and *RbcS*, while it directly represses expression of *S-adenosylmethionine synthetase 1* (*SAM1*)*,* thereby inhibiting ethylene biosynthesis and fruit ripening [25]. SlARF10 also positively regulates chlorophyll biosynthesis by promoting the expressions of *SlGLK1*, *protochlorophyllide oxidoreductase* (*POR*), and *chlorophyll binding protein*s (*CBP*s; Fig. 3). Investigations with transgenic lines indicate that SlARF10 is an activator of starch biosynthesis and sugar metabolism, but the mechanism requires further investigation [33].

**Figure 4:**
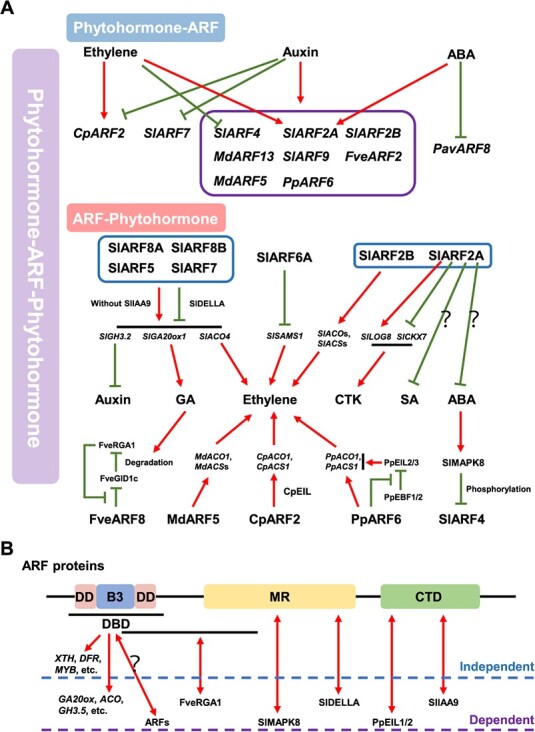
ARFs as important keys in a network of phytohormone TFs that control development and ripening in fleshy fruits. **A** ARF-mediated phytohormone interaction model illustrating the intricate interplay between phytohormones and ARFs in influencing the development and ripening of fleshy fruits. Green lines represent inhibitory actions, while red single-headed arrows indicate activation. Black lines represent all genes involved in the related processes and the black question mark indicates that the regulatory mechanisms are unclear. CTK, cytokinin; SA, salicylic acid; LOG, LONELY GUY; CKX, CYTOKININOXIDASE; MAPK, mitogen-activated protein kinase. **B** Roles of ARF protein domains in regulating hormone-dependent and independent development and ripening of fruits. Red single- and double-headed arrows indicate activation and protein binding/interaction, respectively. Black lines represent the domains of the region involved, and the black question mark indicates that the binding region between ARFs is unclear. DD, dimerization domain; B3, B3 subdomain; DBD, B3-type DNA binding domain; MR, middle region; CTD, carboxy-terminal dimerization domain. The abbreviations for other genes or proteins in (**A**) and (**B**) are as defined in Figs 2 and 3.

Roles of *ARF*s in ripening have also been described in other fleshy fruits. CpARF2 has been verified to promote the ripening of the climacteric fruit papaya (*Carica papaya*) via enhancing ethylene production [34], which is similar to the role of its homologous gene *SlARF2*s in tomato fruit [28]. In this process, it can interact with EIN3/Ethylene Insensitive3-Like1 (CpEIL1), an important ethylene signal transcription factor, to increase the expressions of *CpACS1* and *CpACO1*. Moreover, CpARF2 also can directly accelerate fruit softening via activating cell wall metabolism genes, including *XTH12* and *pectin methylesterase 51* (*PE51*; Fig. 3). However, FveARF2 can directly repress the expressions of *sucrose transporter gene 1* (*SUT1*) and *chalcone synthase* (*CHS*) to suppress, respectively, sugar and anthocyanin accumulation in strawberry fruit [35]. Moreover, MdARF2 has been found to repress the development of red color in the climacteric fruit apple (*Malus* × *domestica*), by inhibiting anthocyanin biosynthetic genes [36], an action which is different from that of its homologs, SlARF2s and CpARF2, in ripening tomato and papaya (Fig. 1). These results suggest that the functions of *ARF2* homologous genes in fleshy fruit ripening are not precisely conserved. In apple fruit, MdARF13, a SlARF4 homolog (Fig. 1), has also been found to repress anthocyanin biosynthesis by directly repressing *dihydroflavonol 4-reductase* (*DFR*), a key gene in the anthocyanin biosynthetic pathway (ABP), and interacting with MdMYB10, an activator of ABP, to suppress its function [37]. In sweet cherry (*Prunus avium*) fruit, PavARF8, which is closely related to ARF3 (Fig. 1), positively regulates *DNA-binding with One Finger 2* (*Dof2*) and *15* (*Dof15*) to suppress fruit softening genes encoding cell wall-modifying enzymes [38]. Moreover, in apple, auxin-induced MdARF5, a SlARF5 homolog (Fig. 1), directly binds to the AuxRE elements of the *MdACS3a*, *MdACS1*, and *MdACO1* promoters to upregulate their expression, leading to enhanced ethylene biosynthesis, which promotes fruit ripening [39] (Fig. 3). Similarly, the homolog of tomato SlARF6A, auxin-activated PpARF6 (Fig. 1), also positively controls peach (*Prunus persica*) fruit ripening by upregulating the expression of ethylene biosynthetic genes, including *PpACS1* and *PpACO1*, to promote ethylene production (Fig. 3). PpARF6 also competes with EIN3-binding F-box protein-1 and -2 (EBF1/2) for binding to ethylene-insensitive3-like proteins-2 and -3 (PpEIL2 and PpEIL3), which activates a positive feedback loop that further enhances ethylene biosynthesis [40].

## ARFs may regulate more important quality attributes of ripening fleshy fruits

Existing evidence shows that ARFs not only regulate fleshy fruit development, including fruit set [21–23], fruit growth [19–26], and seedlessness [19–23], but also control aspects of ripening and development of quality attributes, which includes the biosynthesis or metabolism of starch [31, 33], sugar [25, 33, 35], chlorophyll [25, 31, 33], AsA [32], anthocyanin [35–37], and the cell wall [34, 38] (Figs 2 and 3). ARFs exhibit positive or negative regulatory effects on ripening and development, and bidirectional regulation (i.e. they possess both positive and negative functions) in development. So far, there have been no reports of ARF Class Ib members being involved in these processes (Fig. 1). Although many ARFs have been found to participate in ripening, the roles of ARFs in determining other important quality attributes, such as flavonoids, carotenoids, and volatile aromatic compounds, still lack clear evidence and additional research is required. Detailed functional studies of ARFs during development have mainly been confined to the model climacteric and non-climacteric fruits, tomato and strawberry, where stable transgenic systems have been established, although this has now also been achieved in apple and kiwifruit. Additionally, transient transgenic expression systems have also proved suitable for investigating functions of genes during late stages of development in multiple other fleshy fruits. Nevertheless, successful establishment of additional stable transgenic systems in other fruits will be advantageous.

**Table 1:** Roles of different ARFs and phytohormones involved in fleshy fruit development and ripening.

$\includegraphics{\bwartpath uhae209t1}$

ARFs on green and yellow backgrounds represent genes involved in fleshy fruit development and ripening, respectively. Blue and orange colors indicate *ARF*s regulated by auxin or other phytohormones, whereas a gray background indicates no reported role for phytohormones in regulation of a particular *ARF*.

## ARFs as important keys in a network of phytohormone transcription factors

Phytohormones are the most important upstream regulators governing expression of themselves signaling genes [2]. *ARF* transcript levels are not only positively or negatively regulated by auxin but also by other phytohormones (e.g. ethylene and ABA) in fleshy fruit (Fig. 4A; Table 1). Thus, at least three, and probably more, phytohormones control fruit development and ripening, which implies a phytohormone–ARF interacting pathway. At present, it is not known whether half the reported ARFs, such as *SlARF5*, *SlARF8*s, and *MdARF2*, are regulated by auxin or other phytohormones, although understanding how they are regulated will be a most crucial step. Moreover, *ARF*s also can control phytohormone metabolism (e.g. GA in development and ethylene or ABA in ripening) to influence fleshy fruit development and ripening (Fig. 4A). In addition to ethylene, CTK, auxin, and GA [22, 25, 28, 29, 34, 39, 40], the specific mechanisms of ABA and SA [29] effects on metabolisms mediated by ARFs are still lacking. Additionally, it has been found that several ARFs interact with auxin and other phytohormone signaling proteins to perform their functions, such as SlARFs-SlDELLA [21], FveARF8-FveRAG1 [26], and CpARF2-CpEIL [34] (Fig. 4A). These findings indicate that there is a sophisticated phytohormone–ARF–phytohormone interaction network that governs fleshy fruit development and ripening.

Additionally, auxin can enhance the expression of some *ARF*s to promote ethylene production, which accelerates ripening in climacteric fruits, including tomato, apple, peach, and papaya (Fig. 3). However, SlARF6A can repress tomato fruit ripening by inhibiting ethylene biosynthesis [25], suggesting auxin and ARF members may have both positive and negative roles (i.e. they may be bidirectional regulators) in non-climacteric fruit ripening. Furthermore, it has been observed that auxin has the ability to suppress the ripening of non-climacteric fruits through inhibiting ABA biosynthesis, which is a dominant factor in climacteric fruits [2, 27]. It is possible that auxin represses ABA biosynthesis to inhibit non-climacteric fruit ripening through ARFs. Further research is needed to understand possible differences in auxin-modulated ARFs in climacteric and non-climacteric fruits.

## Roles of ARF structural domains in regulation of fleshy fruit development and ripening by phytohormone-dependent and independent pathways

In fleshy fruit, ARFs exert their functions through phytohormone-dependent and -independent mechanisms. The first mechanism regulates processes by controlling phytohormone metabolism or interacting with other signaling proteins, while the latter involves interactions with transcription factors or directly manipulates the expression of genes involved in the formation of fruit traits (Figs 2 and 3). Their three domains contribute to these two pathways, with each domain performing distinct or overlapping functions (Fig. 4B). The ARFs directly bind to the AuxRE elements of genes involved in fruit traits (phytohormone-independent) or phytohormone biosynthetic and signaling genes (phytohormone-dependent) via their DBD domains. In both mechanisms, several ARFs have been confirmed to interact with transcription factors (e.g. MdMYB10 [37]) and phytohormone signaling proteins in fleshy fruit. Three types of interactive regions in ARF proteins have been identified: (i) the interactive region includes both DBD and MR domains, such as FveRAG1-FveARF8 in strawberry fruit [26]; (ii) the interaction only involves the MR domains, such as SlMAPK8-SlARF4 and SlDELLA-SlARFs in tomato fruit [21, 32]; and (iii) the function of the CTD domain is to bind to the auxin signaling protein AUX/IAA but it also may interact with other proteins. For example, there is a binding interaction between PpEIL1/2 and the C-terminal region containing the CTD domain of PpARF6 in peach fruit [40]. Additionally, ARFs can also bind to each other via their dimerization domains (DDs), which are parts of the DBD domains flanking a B3 subdomain [41]. The dimerization between ARFs may play a significant role in the regulation of fleshy fruit development and ripening, as evidenced by studies such as those on SlARF7-SlARFs [21], SlARF2A-SlARF2A [29], and CpARF2-CpARF2 [34] (Fig. 4B). However, the function of specific binding regions within the DBD domains and how dimerization is regulated and affects development and ripening needs clarification.

## Conclusions and prospects

With an improved understanding of ARFs, we realize that they are involved in regulating fleshy fruit development and ripening in complex and diverse ways, involving interactions with multiple other phytohormones. Future research needs to consider and address the following aspects. What are the differences in auxin-regulated *ARF*s that control ripening between climacteric and non-climacteric fruits? Are Class Ib members functioning as regulators? How do other phytohormones regulate ARF expression, and are there any additional phytohormones participating in this process beyond those currently reported? Are there specific amino acid sequences involved in recognition of other interacting proteins? What are the functions of ARF–ARF dimerization? Further exploration of these questions will enhance our understanding of the various functions and multiple regulatory mechanisms of ARFs, leading to a more comprehensive understanding of the mechanisms and regulatory networks governing fleshy fruit development and ripening.
